# Morphological Control of TiO_2_ Supports for Enhanced Pt‐Catalyzed Methylcyclohexane Dehydrogenation

**DOI:** 10.1002/smsc.202500652

**Published:** 2026-04-19

**Authors:** Yujung Jung, Najwa Fatimah Somanjaya, Hyunsik Hwang, Yoondo Kim, Hyangsoo Jeong, Yongmin Kim, Keunsoo Kim, Minseok Bae, Suk Woo Nam, Muhammad Ridwan, Wangyun Won, Hyuntae Sohn

**Affiliations:** ^1^ Center for Hydrogen and Fuel Cell Research Korea Institute of Science and Technology Seoul Republic of Korea; ^2^ Department of Chemical and Biological Engineering Korea University Seoul Republic of Korea; ^3^ Department of Chemistry University of Indonesia Depok Indonesia; ^4^ Department of Energy and Environmental Engineering KIST School University of Science and Technology Seoul Republic of Korea

**Keywords:** facet engineering, hydrogen production, liquid organic hydrogen carrier (LOHC), methylcyclohexane (MCH), titania

## Abstract

Efficient and durable catalysts are essential for the practical implementation of dehydrogenation processes in liquid organic hydrogen carrier (LOHC) systems. In this study, we demonstrate that the catalytic performance of platinum (Pt)/TiO_2_ catalysts can be fundamentally modified via morphology‐driven facet engineering of the TiO_2_ support for dehydrogenation of methylcyclohexane (MCH). Anatase TiO_2_ supports with distinct exposed facets were synthesized for a direct comparison between {001}‐exposed nanosheets and {101}‐dominant nanorhombic and polyhedral structures. Among these, the Pt catalyst supported on TiO_2_ nanosheets exhibits significantly enhanced catalytic activity and stability during MCH dehydrogenation. Comprehensive spectroscopic and adsorption analyses reveal that the two‐dimensional nanosheet architecture induces strong interfacial electronic interactions, stabilizes metallic Pt species, and promotes favorable surface acidity. This architecture also improves reactant adsorption and accessibility of active sites. Consequently, the Pt/nanosheet‐TiO_2_ catalyst delivers superior intrinsic activity and excellent morphological stability under reaction conditions. This study highlights support morphology and facet exposure as key design parameters to control interfacial chemistry and catalytic functionality, providing a general strategy to advance the development of LOHC‐based hydrogen storage technologies.

## Graphical abstract

This study shows how tailoring TiO_2_ support morphology, particularly through {001}‐exposed nanosheets, reshapes Pt–support interactions during methylcyclohexane dehydrogenation. The nanosheet architecture strengthens electronic coupling, maintains highly dispersed Pt, and improves access to active sites. As a result, Pt/nanosheet‐TiO_2_ catalysts demonstrate elevated activity and stability, offering a practical design route for advanced LOHC dehydrogenation systems.

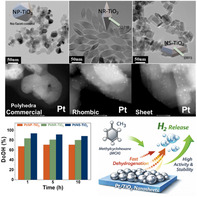

## 1 Introduction

In the transition to an economy based on sustainable energy, hydrogen (H_2_) is considered the ultimate energy carrier owing to its high gravimetric energy density (141.82 MJ kg^−1^) and environmentally friendly combustion [1, 2]. However, realizing a practical hydrogen economy remains challenging because of the lack of safe and efficient infrastructure as well as the extreme conditions required for hydrogen storage, such as ultra‐high pressures (350–700 bar) and cryogenic temperatures (20 K) [3]. To address these challenges, liquid organic hydrogen carriers (LOHCs) have emerged as a promising alternative [4]. LOHC technology facilitates safe storage of hydrogen under mild, ambient conditions while offering a high volumetric hydrogen density comparable to that of liquid hydrogen. Among various LOHC systems, the methylcyclohexane (MCH)–toluene (TOL) system is considered a leading candidate owing to its high hydrogen storage capacity (6.2 wt%), favorable physical properties, low toxicity, and compatibility with existing fuel infrastructure [5, 6].

The core of MCH‐TOL technology lies in the endothermic dehydrogenation of MCH (C_7_H_14_ → C_7_H_8_ + 3H^2^; Δ*H*° = 205 kJ mol^−1^) [7]. Therefore, developing highly active and stable catalysts is essential to drive this reaction with high energy efficiency at relatively low temperatures [8]. Platinum (Pt) is widely recognized as the benchmark catalyst for MCH dehydrogenation owing to its exceptional intrinsic activity for selective C—H bond cleavage [9]. Conventionally, Pt is dispersed on high‐surface‐area supports such as gamma‐alumina (γ‐Al_2_O_3_), and these catalysts exhibit high initial MCH conversion at reaction temperatures of 300°C–350°C [10, 11, 12]. Despite their high initial activity, these catalysts often undergo rapid deactivation under high‐temperature reaction conditions because coke deposition progressively blocks the active sites and disrupts the hydrogen release pathway [13]. For example, Okada et al. reported that conventional Pt/Al_2_O_3_ catalysts suffer extreme deactivation, primarily attributed to the coke formation on the acidic sites of the support [14]. Furthermore, Nataliia Marchenko et al. observed that the strong acid sites on the Al_2_O_3_ support can trigger undesirable side reactions, and that the supported Pt nanoparticles themselves exhibit a high ratio of low‐coordination sites that contribute to byproduct formation [15]. Consequently, catalyst deactivation remains a major obstacle to the commercialization of Pt‐based catalysts.

To address these stability issues, numerous studies have explored modulating the electronic properties of Pt employing various strategies, such as the introduction of promoters, formation of bimetallic alloys, and modification of support materials [16]. Among these approaches, employing a reducible oxide support has garnered significant attention as a means to address the limitations of conventional supports [17]. Notably, Sugiura et al. demonstrated that the addition of a trace amount of TiO_2_ to an Al_2_O_3_ support effectively modified the electronic state of Pt and prevented methane formation from further toluene decomposition while inhibiting coke formation, thereby enhancing the selectivity and durability of the catalyst [18]. Moreover, Sekine et al. attributed the high stability of Pt/TiO_2_ catalysts to strong metal–support interaction (SMSI) [19]. They elucidated that this interaction facilitates electron transfer from the TiO_x_ support to the Pt nanoparticles and creates electron‐rich active sites. Consequently, the π‐back‐donation to the aromatic ring of toluene is suppressed, which weakens product adsorption and effectively inhibits catalyst deactivation. Recent studies have extensively demonstrated that optimizing such metal–support interactions is a crucial strategy for maximizing the durability of various Pt‐based catalysts [20, 21]. Furthermore, strategically engineering site‐to‐site interactions has also been proven to significantly boost overall catalytic performance [22, 23]. Therefore, reducible oxide supports like TiO_2_ have garnered significant attention for their potential to enhance catalyst performance.

Meanwhile, the nature and strength of this interaction can be significantly influenced by the crystal facets exposed on the support surface [24]. This has spurred research into the “facet engineering” of catalyst supports like anatase TiO_2_. Byun et al. demonstrated that the morphological engineering of TiO_2_ can substantially impact Pt dispersion and promote strong interfacial coupling [25]. Similarly, Wu et al. showed that Pt dispersion, which is governed by the intensity of metal–support interactions, can be modulated by the morphology of TiO_2_ [26]. In another systematic study, Liu et al. investigated the crystal facet effect of TiO_2_ supports for the selective hydrogenation of nitroarenes over Pd/TiO_2_ catalysts [27]. They reported that TiO_2_ supports with dominant {101} facets generated more oxygen vacancies and promoted uniform Pd particle dispersion compared with those with {001} facets, resulting in substantially improved activity and selectivity. Although prior studies have established the benefits of modifying supports, the fundamental relationship between specific TiO_2_ crystal facets, the resulting interfacial electronic state, and catalytic performance in MCH dehydrogenation remains unclear [28]. Therefore, precisely controlling the support morphology at the atomic level presents a key strategy to optimize catalyst performance by directly tuning the dispersion and electronic properties of Pt.

In this study, we investigate the pivotal role of TiO_2_ support morphology in regulating the catalytic behavior of Pt nanoparticles for MCH dehydrogenation. Anatase TiO_2_ supports with well‐defined surface facets were synthesized in three representative morphologies: {001}‐faceted nanosheets (NS), {101}‐dominant rhombic particles (NR), and polyhedral anatase TiO_2_ with random facet orientations (NP). Following Pt deposition, the catalysts were systematically compared in terms of catalytic performance and physicochemical properties. Our findings demonstrate that TiO_2_ facet engineering is not merely a structural modification, but rather serves as a key lever to tune the SMSI state and thus govern the activity and stability of the catalyst.

## 2 Experimental

### 2.1 Materials

Oleic acid (90%), oleylamine (70%), tert‐butanol (≥99%), benzyl alcohol (≥99%), titanium (IV) oxide, anatase (≥99.5%), and titanium (IV) fluoride and chloroplatinic acid solution (H_2_PtCl_6_, 8 wt% in H_2_O Pt) were purchased from Sigma–Aldrich. Titanium (IV) butoxide (≥99%) was purchased from Alfa Aesar. MCH was supplied by Samchun Pure Chemicals (Korea). Anhydrous ethanol (99%) was purchased from Daejung (Korea), and deionized water (18.2 MΩ·cm) was obtained from a Milli‐Q system.

### 2.2 Catalyst Synthesis

#### 2.2.1 Synthesis of Support Materials

**FIGURE 1:**
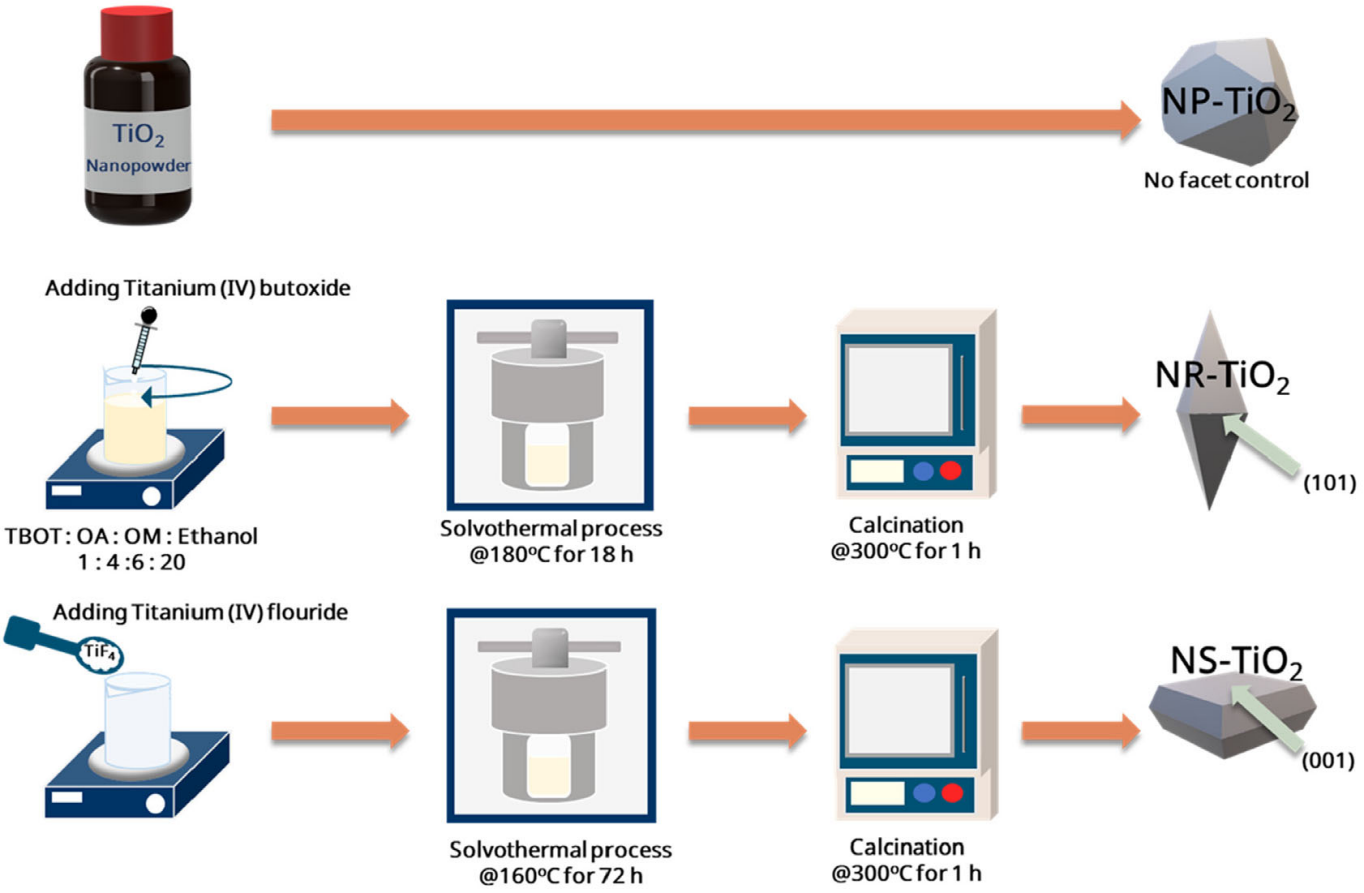
Schematic of facet‐controlled synthesis routes for anatase TiO_2_ with different morphologies.

The overall synthesis procedures for the TiO_2_ supports and Pt/TiO_2_ catalysts are illustrated in Figure 1. Three types of TiO_2_ supports were utilized in this study, including a purchased commercial TiO_2_ support designated as nanopolyhedral (NP), and two synthesized supports, a rhombic dodecahedra‐shaped support designated as nanorhombic (NR) and a sheet‐shaped support designated as NS. The NR support was synthesized via a solvothermal method using anhydrous ethanol as the solvent [29]. Oleic acid and oleyl amine were dissolved in ethanol and stirred for 30 min. Subsequently, titanium butoxide was added as the titanium precursor, resulting in a final molar ratio of approximately 1:4:6 for titanium butoxide, oleic acid, and oleylamine, respectively. The mixture was transferred to a 50‐mL Teflon‐lined stainless‐steel autoclave and heated at 180°C for 18 h. The precipitate was collected via centrifugation, washed with 99% ethanol, and dried under vacuum at ambient temperature overnight. Calcination was performed at 300°C for 1 h with a ramp rate of 5°C min^−1^ to yield the final NR support. The NS support was synthesized via a solvothermal method using tert‐butanol and benzyl alcohol as solvents [30]. Titanium (IV) tetrafluoride was added as the titanium precursor. The mixture was transferred to a 500‐mL Teflon‐lined stainless‐steel autoclave and maintained at 160°C for 72 h. After cooling to room temperature, the precipitate was collected, washed thoroughly with 99% ethanol, and dried at 100°C overnight. The dried powder was then calcined at 300°C for 1 h with a ramp rate of 5°C min^−1^ to yield the final NS support.

#### 2.2.2 Pt Deposition on TiO_2_ Supports

Platinum nanoparticles were deposited onto the three TiO_2_ supports (NP, NR, and NS) via the incipient wetness impregnation method [31]. Prior to impregnation, the supports were pre‐dried at 100°C to remove residual moisture. The total volume of chloroplatinic acid hexahydrate solution (8 wt% Pt) required to achieve a final Pt loading of 1 wt% was first calculated. This specific loading was kept constant across all catalysts to ensure that the effects of TiO_2_ morphology and facet exposure could be systematically compared under the same metal content. The precursor solution was diluted to a total volume equivalent to three times the total pore volume of the support, and the solution was added to the supports in three successive portions, each equivalent to the pore volume of the support. After each addition, the mixture was thoroughly mixed to ensure uniform distribution of Pt. Following impregnation, the samples were dried at 100°C overnight and subsequently calcined at 350°C for 1 h with a ramp rate of 5°C min^−1^.

### 2.3 Catalyst Characterization

Inductively coupled plasma‐optical emission spectrometry (ICP‐OES) was employed to determine the actual Pt loading on the catalysts. Powder X‐ray diffraction (XRD) patterns were recorded on a Bruker D8 ADVANCE diffractometer (Cu Kα radiation, 40 kV, 200 mA) over a 2*θ* range of 20°–80° at 6° min^−1^. Transmission and scanning transmission electron microscopy (TEM/STEM), including high‐angle annular dark‐field (HAADF) imaging, were conducted on an FEI Titan 80–300 microscope at 300 kV. Samples were prepared by ultrasonically dispersing the powder in ethanol and drop‐casting onto a carbon‐coated copper grid. Raman spectra were acquired on a Renishaw InVia Raman Microscope via 532 and 785 nm laser excitation. X‐ray photoelectron spectroscopy (XPS) was performed on a Thermo Scientific K‐Alpha+ system with a monochromatic Al Kα source. Binding energies were calibrated to the adventitious C 1s peak (284.8 eV), and spectra were fitted using the Avantage software. Nitrogen adsorption–desorption isotherms were measured at −196°C on a Micromeritics 3Flex analyzer. Samples were degassed under vacuum at 200°C for 4 h prior to analysis.

Ammonia temperature‐programmed desorption (NH_3_‐TPD) was performed using a BELCAT‐M analyzer (MicrotracBEL Corp.). Approximately 50 mg of the sample was pre‐reduced in a 10% H_2_/He stream flowing at 50 mL/min at 350°C for 1 h. After cooling to 100°C under a He flow, the sample was saturated with pure NH_3_ introduced at a flow rate of 50 mL/min for 30 min. Subsequently, the sample was purged with a He carrier gas at 30 mL/min for over 1 h to completely remove physisorbed ammonia and stabilize the thermal conductivity detector (TCD) signal. The TPD profile was then recorded while heating from 100 to 800°C at a ramp rate of 10°C/min under a constant He flow of 30 mL/min. Temperature‐programmed oxidation (TPO) of the spent catalysts was also performed on the same instrument to evaluate carbon deposition. The spent sample was first purged with a He flow at 200°C for 1 h to remove residual volatile and physisorbed species. After cooling to 30°C, the TPO profile was recorded by heating the sample to 750°C at a ramp rate of 10°C/min under a 5% O_2_/He stream flowing at 30 mL/min.

CO pulse chemisorption was conducted on the same instrument following an identical pre‐reduction procedure. After cooling to the measurement temperature, CO pulses were injected into a He carrier stream flowing at 30 mL/min until saturation was confirmed. The metallic dispersion was calculated assuming a CO:Pt stoichiometry of 1:1.

In situ diffuse reflectance infrared Fourier transform spectroscopy (DRIFTS) was employed to investigate the surface species during the reaction using a Nicolet iS50 spectrometer. The sample was prereduced in 10% H_2_/Ar stream flowing at 20 mL/min at 350°C for 1 h. Following reduction, the sample temperature was adjusted to 320°C. A background spectrum was collected after the temperature stabilized. MCH vapor was then introduced by bubbling an Ar carrier gas flowing at 20 mL/min through liquid MCH for 30 min. Subsequently, the gas flow was switched to a pure Ar stream flowing at 20 mL/min to purge the gas phase. Time‐resolved spectra were recorded continuously at 320°C during this Ar purging phase to monitor the evolution of adsorbed species.

### 2.4 Catalytic Activity Tests

Catalytic dehydrogenation activity was evaluated in a lab‐scale fixed tubular stainless‐steel reactor using 0.1 g of catalyst Pt/NP‐TiO_2_, Pt/NR‐TiO_2_, and Pt/NS‐TiO_2_. Prior to the reaction, the catalyst was pre‐reduced in situ at 350°C for 1 h under a flow of pure H_2_. Following this treatment, the reaction was performed at 320°C with the introduction of liquid‐phase MCH. The reaction was conducted at atmospheric pressure with a co‐feed of MCH and H_2_ (H_2_/MCH molar ratio = 1). The catalytic activity was then evaluated for 10 h at liquid hourly space velocities (LHSV) of 3, 10, and 15 ml_MCH_·g_cat_^−1^·h^−1^. The liquid product (toluene) was condensed from the reactor effluent using a chiller, and the flow rate of the remaining H_2_ gas was quantified using a mass flow meter. The degree of dehydrogenation (DoDH) was then calculated from the net H_2_ production rate via Equation (1), based on the theoretical maximum for complete MCH conversion. The theoretical H_2_ production rate refers to the amount of hydrogen produced assuming 100% dehydrogenation of MCH.

(1) $$\text{Degree of dehydrogenation}\;(\%,DoDH)=\frac{\text{measured}\;H_{2}\;\text{production}}{\text{theoretical}\;H_{2}\;\text{production}}\times 100\%$$

## 3 Results and Discussion

### 3.1 Structural Characterization of TiO_2_ and Pt/TiO_2_

**FIGURE 2:**
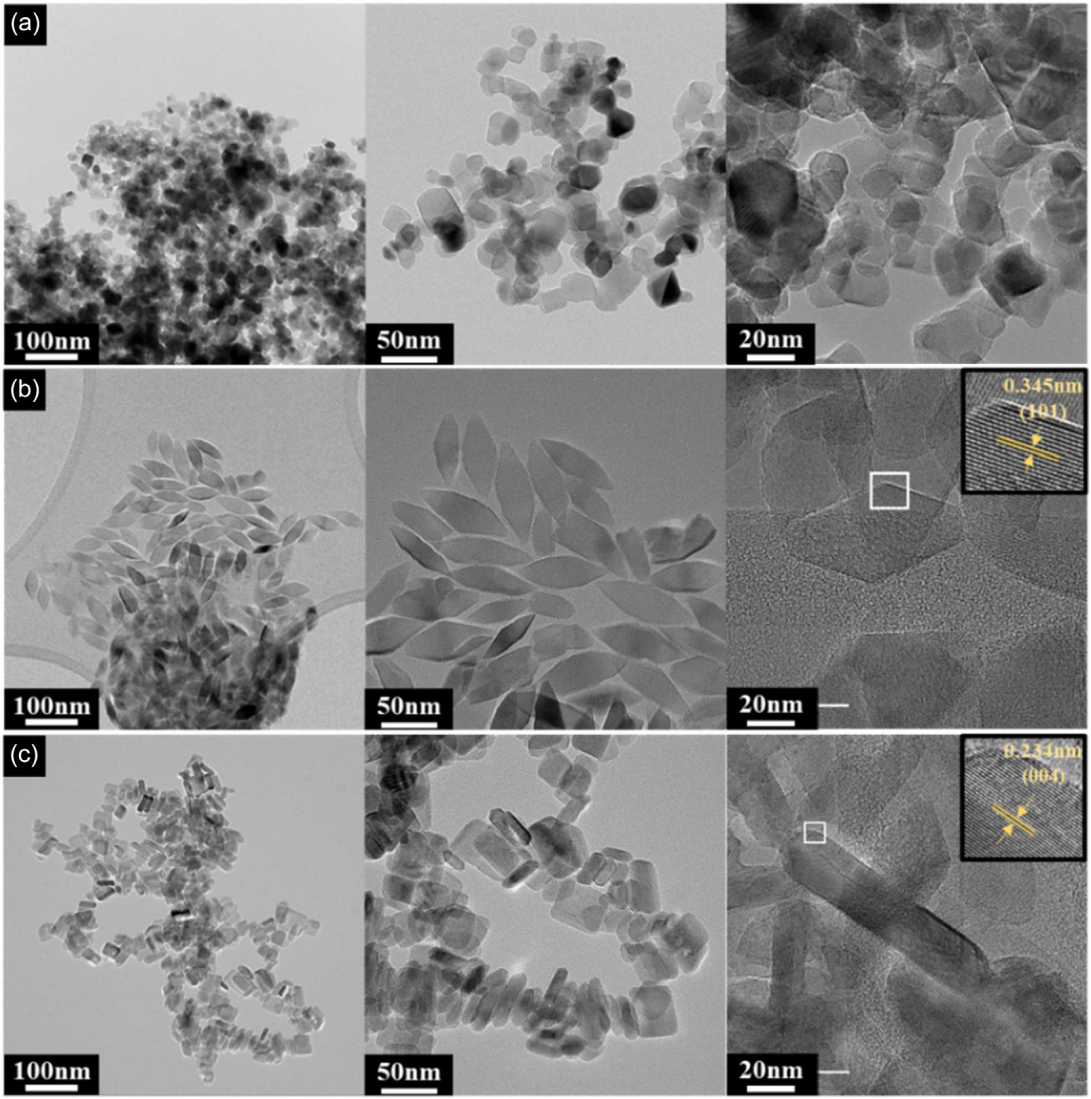
TEM images showing the morphologies of the as‐prepared NP‐TiO_2_ (a), NR‐TiO_2_ (b), and NS‐TiO_2_ (c).

Figure 2 presents the TEM images used to investigate the morphologies of the TiO_2_ supports (NP, NR, and NS). The commercially purchased NP support shown in Figure 2a exhibits an irregular polyhedral morphology with particles of diverse sizes, averaging approximately 21 nm. By contrast, the solvothermally synthesized supports in Figure 2b,c feature highly uniform and well‐defined nanostructures, which confirms the efficacy of the proposed synthesis strategy. The NR support in Figure 2b comprises monodisperse rhombic dodecahedra with an average length and width of 58 and 20 nm, respectively. This specific morphology is extensively documented for preferentially exposing the thermodynamically stable anatase {101} facets (d‐spacing = 0.345 nm) [32]. As shown in Figure 2c, the NS support comprises uniform NS with an average lateral dimension of approximately 40 nm and a thickness of ~8 nm. This distinct sheet‐like structure is intentionally designed to maximize the exposure of the high‐energy, and consequently highly reactive {001} crystal facets (d‐spacing = 0.234 nm) [32, 33].

**FIGURE 3:**
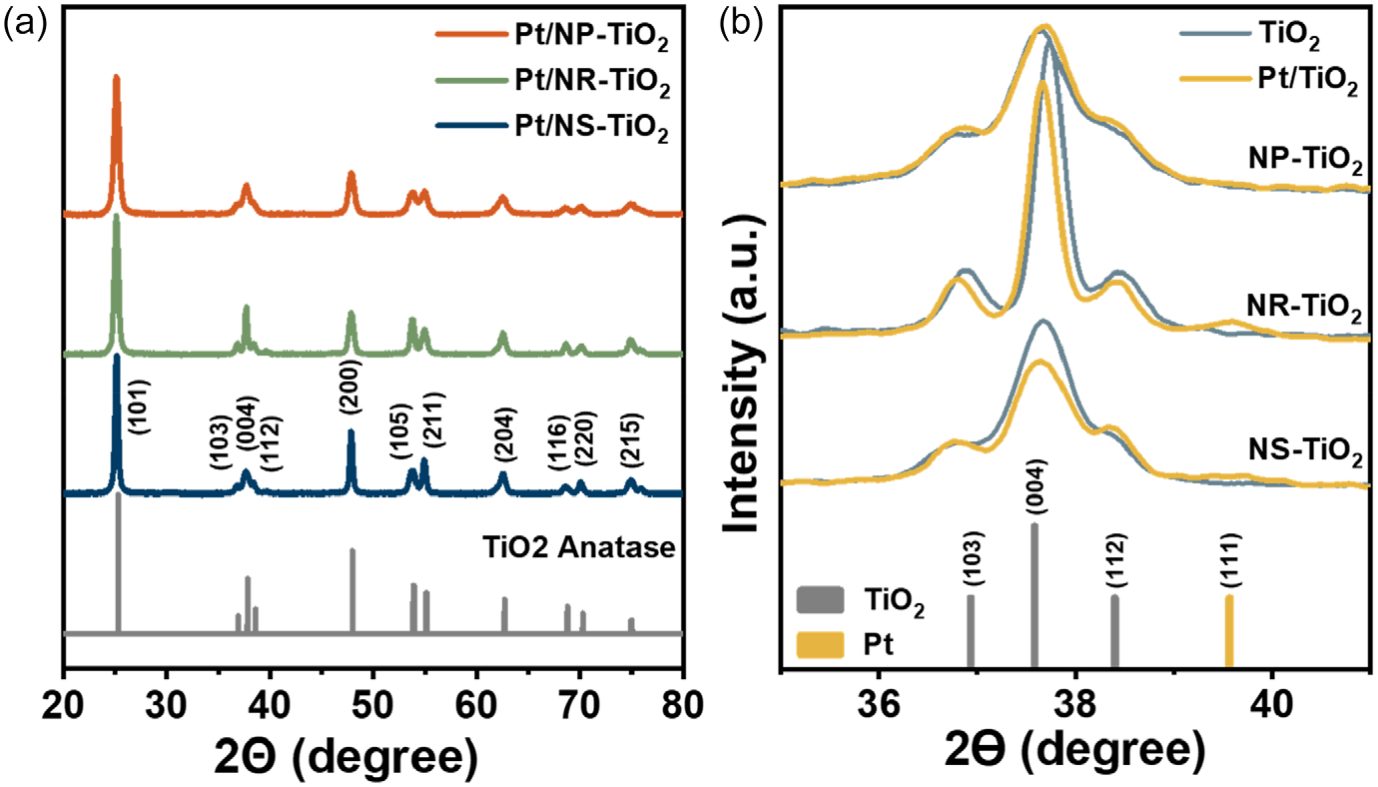
XRD patterns of (a) Pt/TiO_2_ catalysts with different TiO_2_ morphologies and (b) a comparison between bare TiO_2_ supports and the corresponding Pt/TiO_2_ catalysts.

The crystal structure and phase purity of the Pt/TiO_2_ catalysts after addition of Pt were investigated employing XRD, with the results presented in Figure 3. The diffraction patterns for all three samples (Pt/NP‐TiO_2_, Pt/NR‐TiO_2_, and Pt/NS‐TiO_2_) in Figure 3a are exclusively indexed to the pure anatase phase of TiO_2_ (JCPDS No. 21‐1272). No detectable impurity phases were observed, validating that the crystallographic integrity of the supports was effectively maintained without phase transformation even after Pt impregnation and calcination at 350°C. The diffraction patterns for both the Pt/NP‐TiO_2_ and Pt/NR‐TiO_2_ catalysts are characteristic of polycrystalline anatase, exhibiting the {101} reflection (2*θ* = 25.3°) as the highest intensity peak, whereas the {200} reflection (2*θ* = 48.0°) exhibits a lower relative intensity, which is typical for standard anatase powder. The Pt/NS‐TiO_2_ catalyst retained the {101} reflection as the most intense peak while exhibiting a significantly enhanced relative intensity of the {200} reflection compared with the other samples. This altered {200}/{101} intensity ratio reflects the preservation of the NS structure [34, 35]. The {004} reflection at 2*θ* = 37.8° exhibits pronounced broadening, which is attributed to the limited crystallite dimension along the {001} (c‐axis) direction and is consistent with the NS thickness of ~8 nm observed by TEM. By contrast, the {200} reflection remained prominently sharp, which indicates high lattice coherence along the {100}/{010} directions and aligns closely with the large lateral size (~40 nm) of the NS. Collectively, these anisotropic diffraction features confirm that the unique NS morphology of the NS support is preserved after Pt impregnation. Furthermore, a detailed inspection of the XRD patterns in Figure 3b revealed an additional broad, low‐intensity peak centered around 2*θ* ≈ 39.8°, which corresponds to the {111} plane of metallic platinum [36, 37]. The significant broadening of this peak strongly indicates the formation of ultrasmall and highly dispersed Pt nanoparticles [38].

**FIGURE 4:**
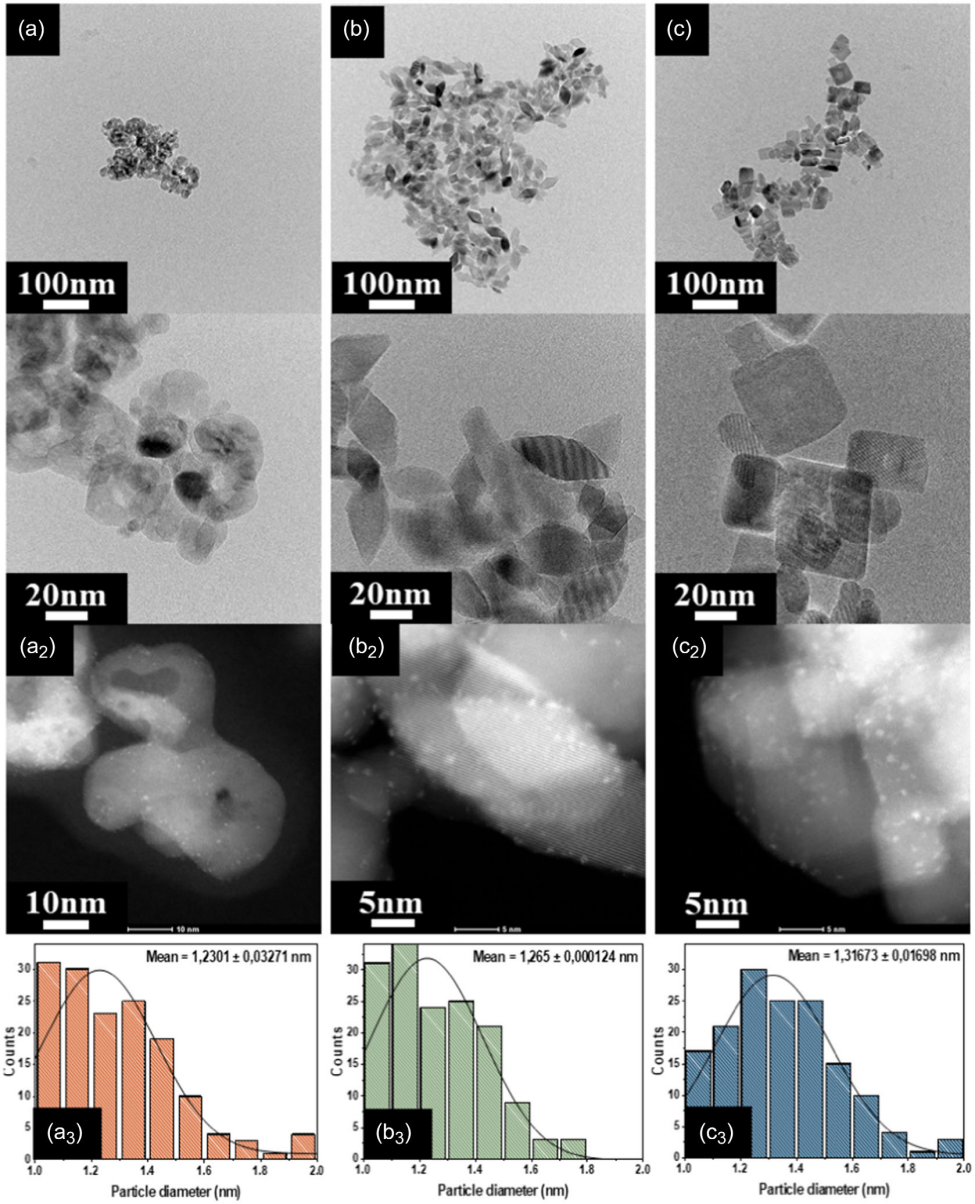
TEM images of calcined (a) NP‐TiO_2_, (b) NR‐TiO_2_, and (c) NS‐TiO_2_ supports; (a_2_–c_2_) HAADF–STEM images of Pt/TiO_2_ catalysts and (a_3_–c_3_) corresponding Pt particle size distribution histograms.

Figure 4 presents the TEM and HAADF–STEM images obtained to verify both the retention of support morphology and the dispersion of Pt nanoparticles after loading. First, the low‐magnification TEM images (Figure 4a–c) confirm that the distinct shapes of the NP, NR, and NS supports were effectively maintained without structural collapse or aggregation, which demonstrates their high thermal stability even after calcination at 300°C. This observation underscores the high thermal stability of the synthesized supports [39]. Prior to further morphological analysis, the actual Pt loading was quantified via ICP analysis (Table 1) where the results confirmed that the Pt content present in each catalyst was identical across all samples (~1 wt.%).

**TABLE 1:** Surface and dispersion properties of Pt/TiO_2_ catalysts.

	Pt, wt%^a^	Dispersion, %^b^	Adsorbed amount, cm^3^/g	BET, m^2^/g	Total pore volume, cm^3^/g	Average pore diameter, nm
Pt/NP‐TiO_2_	1.00	9.95	0.11	64.86	0.18	11.66
Pt/NR‐TiO_2_	1.03	13.67	0.16	62.42	0.15	8.56
Pt/NS‐TiO_2_	1.04	30.76	0.35	49.18	0.22	17.74

a Determined by ICP‐OES.

b Calculated from CO pulse chemisorption data.

The HAADF–STEM images (Figure 4a_2_–c_2_) provide direct evidence of Pt dispersion. In these Z‐contrast sensitive images, Pt nanoparticles are clearly distinguishable as bright nanodots uniformly distributed across the TiO_2_ surfaces, which indicates successful and uniform metal loading. To quantify this distribution, the sizes of the particles were analyzed by measuring over 100 nanoparticles for each sample, and the results are presented in the histograms in Figure 4a_3_–c_3_. The calculated average Pt particle sizes were found to be 1.23 ± 0.03 nm, 1.26 ± 0.01 nm, and 1.31 ± 0.01 nm for Pt/NP‐TiO_2_, Pt/NR‐TiO_2_, and Pt/NS‐TiO_2_, respectively. It is particularly noteworthy that all three catalysts exhibited Pt nanoclusters with remarkably similar average sizes despite their distinct support morphologies and predominantly exposed crystal facets ({101} for NR and {001} for NS) [40]. This observation is crucial because it indicates that the Pt nanoparticle size was not a significant variable in this study, which facilitates a more direct comparison of the facet‐dependent effects of the support on catalytic activity.

### 3.2 Surface and Electronic Properties of Pt/TiO_2_

In heterogeneous catalysis, catalytic performance is primarily governed by the exposure of active metal sites and their electronic state, which is determined by the interaction with the support [41, 42]. Therefore, elucidating the influence of synthesized TiO_2_ morphologies on the surface chemistry and geometric structure is essential to understand the origin of catalytic activity. In this section, CO‐chemisorption, XPS, and Raman spectroscopy were employed to comprehensively analyze the number of active sites as well as the electronic and structural interactions at the Pt‐TiO_2_ interface.

The efficiency of the MCH dehydrogenation reaction is directly proportional to the accessible metallic Pt surface area [43]. The CO pulse chemisorption results, summarized in Table 1, demonstrate that the support morphology significantly influences Pt dispersion. The Pt dispersion increased in the order of Pt/NP‐TiO_2_ < Pt/NR‐TiO_2_ < Pt/NS‐TiO_2_, with the Pt/NS‐TiO_2_ catalyst achieving the highest dispersion of 30.8%. This trend aligns with the Pt particle sizes determined via HAADF–STEM. TEM analysis confirmed that the initial Pt particle sizes were nearly identical across all catalysts, ranging from approximately 1.2 to 1.3 nm. Despite these comparable particle sizes, Pt/NS‐TiO_2_ exhibited a substantially higher CO uptake, which indicates that the two‐dimensional NS support enhances the accessibility of Pt active sites [44]. The planar morphology of the NS support suppressed Pt agglomeration and minimized site blockage leading to more geometrically accessible Pt sites available for reaction.

**FIGURE 5:**
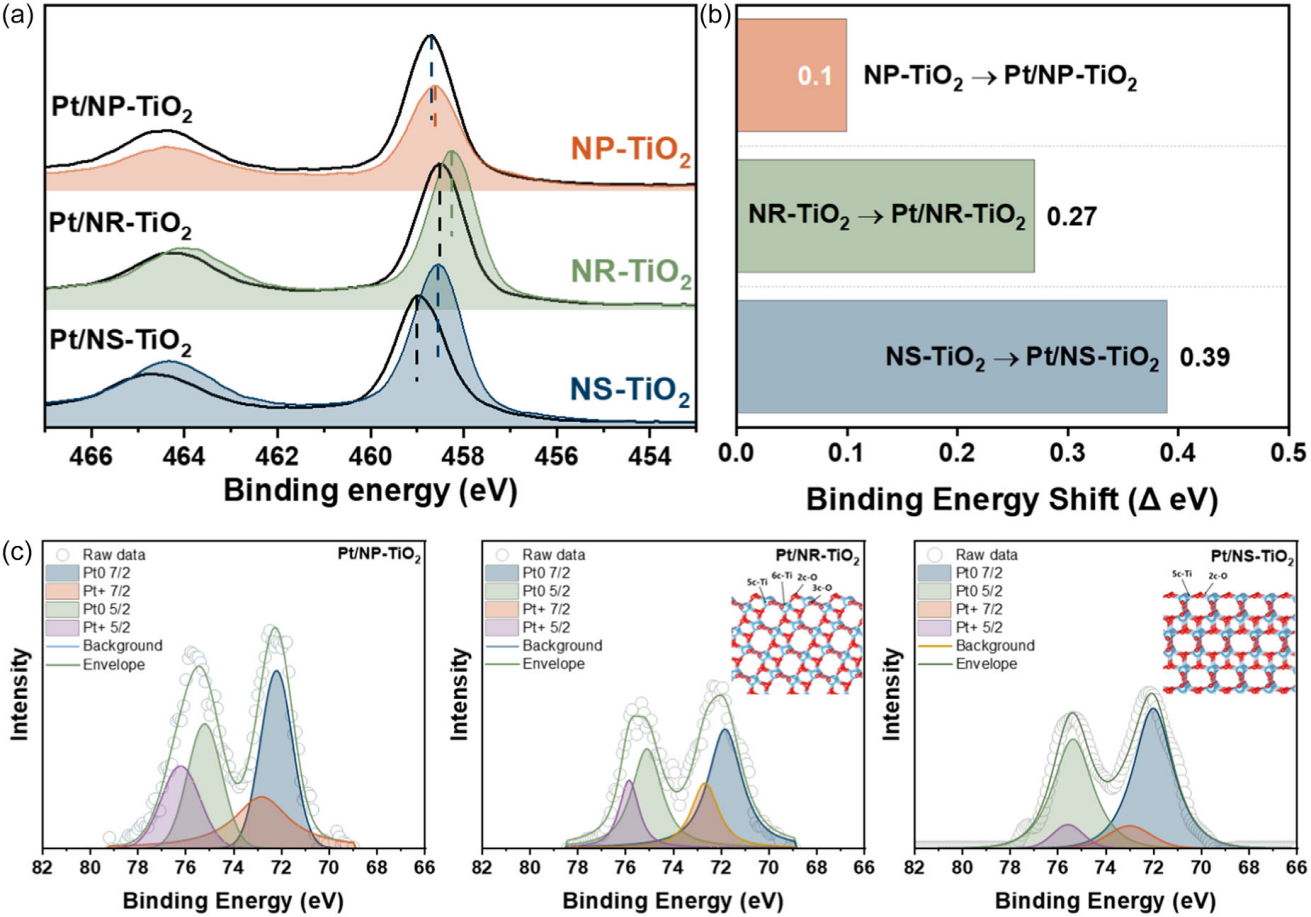
XPS results of TiO_2_ supports and Pt/TiO_2_ catalysts: (a) Ti 2p core‐level spectra, (b) binding energy shift (ΔBE) values, and (c) deconvoluted Pt 4f spectra of Pt/TiO_2_ catalysts.

XPS was performed to investigate the influence of support morphology on the electronic structure of Pt and to elucidate the nature of the metal–support interaction. Figure 5a compares the Ti 2p core‐level spectra before and after Pt impregnation. Following Pt deposition, the Ti 2p peaks for all catalysts exhibited a positive shift in binding energy. This strongly suggests a net electron transfer phenomenon from the Ti atoms of the TiO_2_ support to the Pt nanoparticles [45]. Notably, Figure 5b reveals that the magnitude of this binding energy shift (ΔBE) varied distinctly depending on the support morphology. The Pt/NS‐TiO_2_ catalyst recorded the largest shift of 0.39 eV, which is significantly higher than that of Pt/NR‐TiO_2_ (0.27 eV) or Pt/NP‐TiO_2_ (0.10 eV). This result is attributed to the characteristics of the high‐energy {001} facets predominantly exposed on the NS support. The {001} facets contain an abundance of structurally undercoordinated Ti sites, which makes them chemically more active than other crystal facets and capable of inducing a stronger electronic interaction with Pt [46]. Consequently, our results demonstrate that the {001} facets of the NS are the direct driving force inducing the most profound interfacial coupling. This support‐driven electronic modification effectively altered the chemical state of the Pt metal. Deconvolution analysis of the Pt 4f spectra presented in Figure 5c revealed that the proportion of metallic Pt (Pt^0^), the key active species for dehydrogenation, followed a trend exactly matching the interaction strength. The Pt/NS‐TiO_2_ catalyst exhibited the highest metallicity, with 92% of total Pt species existing in the Pt^0^ state. This implies that intense electron transfer originating from the {001} facets increased the electron density on the Pt surface, which prevented Pt oxidation and contributed to the stable maintenance of the reduced metallic state [47].

**FIGURE 6:**
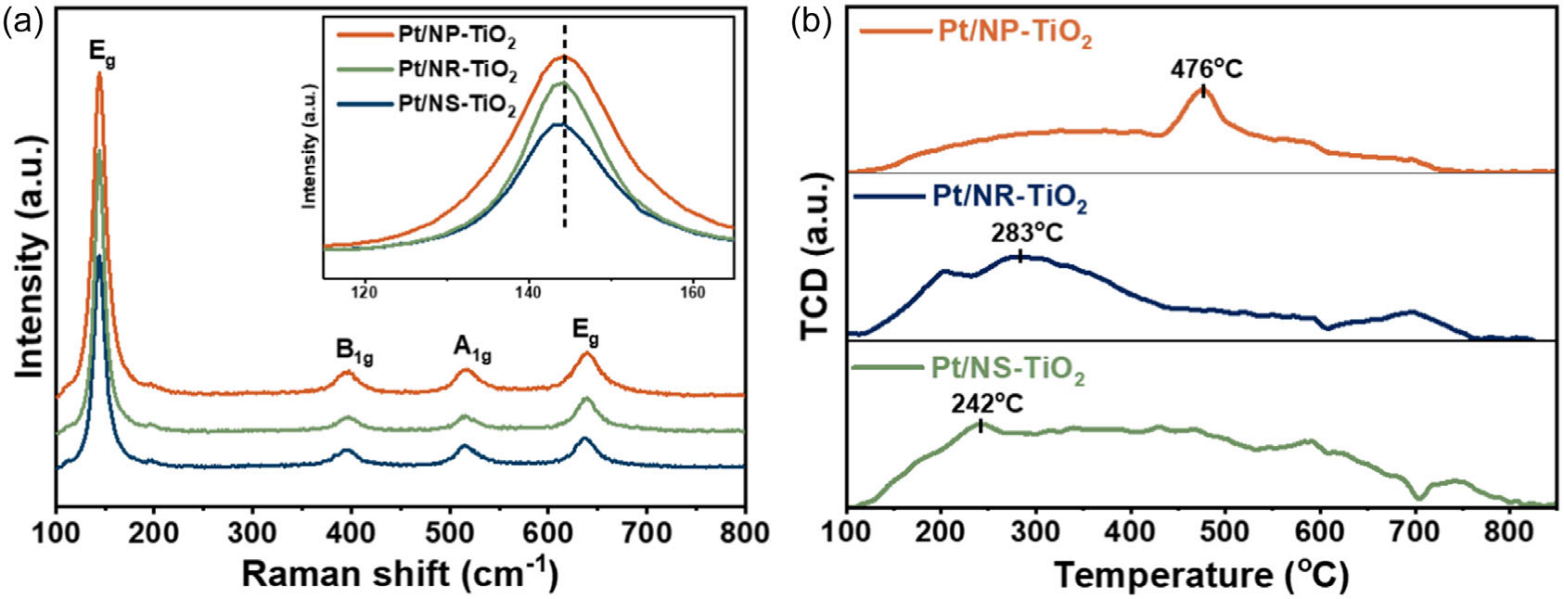
(a) Raman spectra and (b) NH_3_‐TPD Analysis of Pt/TiO_2_ catalysts.

Finally, Raman spectroscopy was employed to cross‐validate whether the electronic interaction was accompanied by structural lattice distortion. In the Raman spectra presented in Figure 6a, the Pt/NS‐TiO_2_ catalyst exhibits the most pronounced red‐shift and asymmetric broadening of the E_g_ mode, the primary peak of anatase TiO_2_. This spectral feature indicates significant lattice strain and defects, such as oxygen vacancies, induced within the TiO_2_ lattice by the strong interaction at the Pt–support interface [34]. Collectively, the XPS and Raman analyses demonstrate that the {001} facets of the NS facilitate the strongest electronic and structural interactions with Pt, which identify this distinct interfacial coupling as the key factor driving the superior performance of the Pt/NS‐TiO_2_ catalyst.

The surface acidity of the catalysts was characterized by ammonia temperature‐programmed desorption (NH_3_‐TPD) as shown in Figure 6b. The Pt/NS‐TiO_2_ catalyst exhibited the highest acid site density according to the NH_3_ desorption peak area, dominated by weak and medium‐strength acid sites, relative to Pt/NR‐TiO_2_ and Pt/NP‐TiO_2_. This observation indicates that Pt/NS‐TiO_2_ provides a maximal concentration of sites favorable for reactant adsorption. This high acid site density directly correlates with the SMSI strength confirmed via XPS. The coordinatively unsaturated Ti sites on the {001} crystal facets possibly serve as the primary Lewis acid sites. This favorable distribution promotes effective interaction of MCH at the metal–support interface, where the cycloalkane ring can be anchored near adjacent Pt active sites, facilitating subsequent C—H bond activation [46, 48]. Notably, the {001} facets provide a higher density of these unsaturated Ti^4+^ sites compared to other morphologies, resulting in stronger Lewis acidity. This structural feature is therefore considered to contribute to the enhanced catalytic activity observed for the Pt/NS‐TiO_2_ catalyst.

### 3.3 Activity Results of Pt/TiO_2_ Catalysts

**FIGURE 7:**
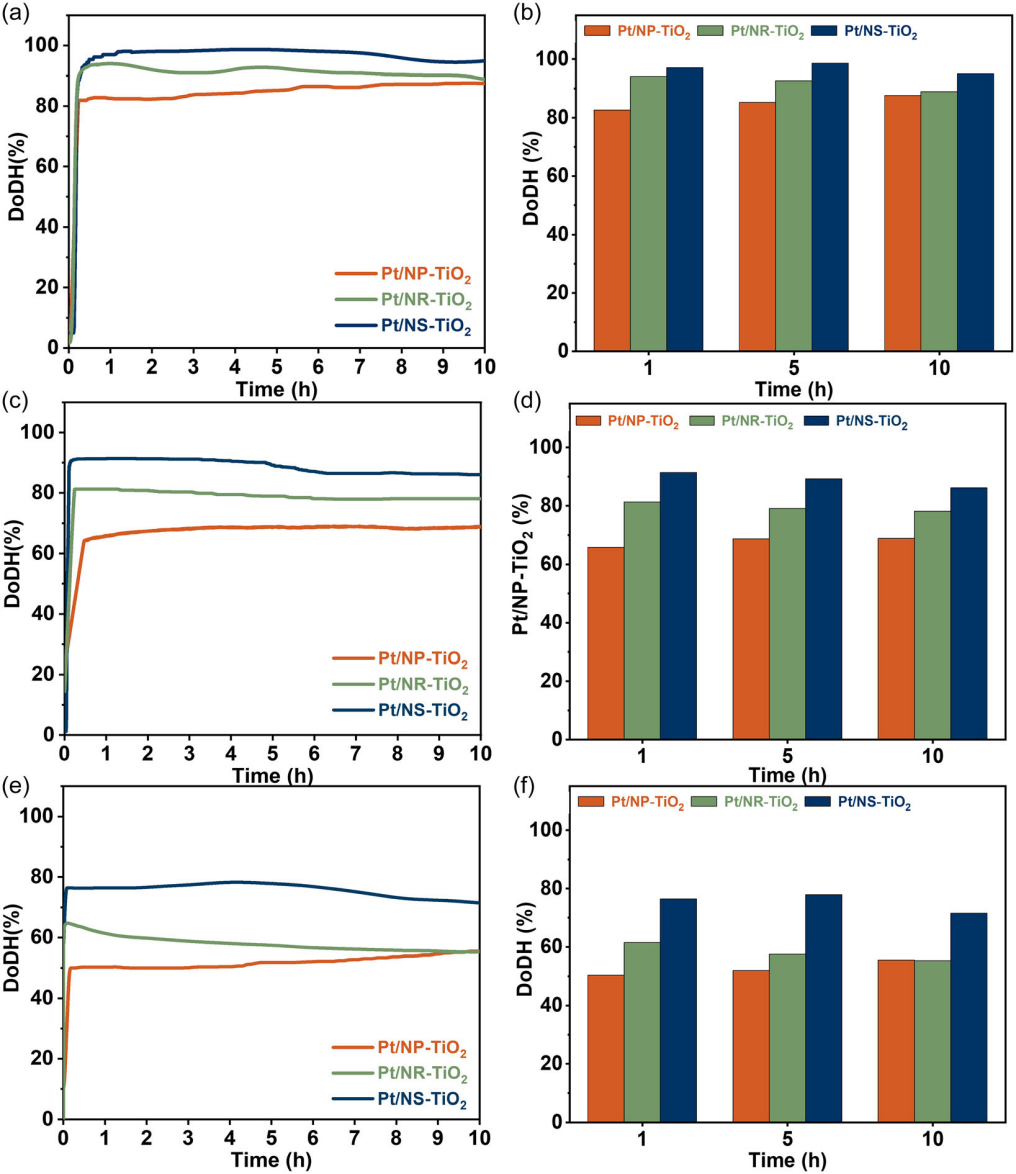
DoDH of Pt/TiO_2_ catalysts at different LHSV of 3 (a,b), 10 (c,d), and 15 h^−1^ (e,f).

The catalytic performance of the Pt/TiO_2_ catalysts for MCH dehydrogenation was evaluated at multiple temperatures and LHSV (LHSV = 3, 10, and 15 h^−1^), as presented in Figure 7. A consistent activity trend was observed across all conditions: Pt/NP‐TiO_2_ < Pt/NR‐TiO_2_ < Pt/NS‐TiO_2_. The Pt/NS‐TiO_2_ catalyst exhibited the highest conversion, reaching 99.7% at 320°C (LHSV = 3 h^−1^), and maintained superior performance even at a high LHSV of 15 h^−1^. By contrast, Pt/NR‐TiO_2_ showed intermediate performance, whereas Pt/NP‐TiO_2_ displayed the lowest activity. GC analyses of both liquid and gaseous products confirmed that toluene was the only detectable product, with no observable formation of other byproducts such as methane under the present reaction conditions.

This distinct activity trend strongly correlates with the physicochemical properties discussed earlier. Specifically, the superior activity of Pt/NS‐TiO_2_ is attributed to its highest fraction of metallic Pt^0^ species, greatest density of weak acid sites, and optimal Pt dispersion. Conversely, the intermediate performance of Pt/NR‐TiO_2_ corresponds to its well‐defined bipyramidal morphology with moderate dispersion, whereas the inferior activity of Pt/NP‐TiO_2_ stems from its irregular nanopolyhedra structure and nonuniform Pt distribution.

Fundamentally, these performance differences result from morphology‐dependent variations in interfacial electronic coupling. Unsaturated surface sites on the metal oxide support act as electron donors, enhancing charge transfer to metal nanoparticles. This mechanism intensifies the metal–support interaction, facilitating Pt dispersion and the stabilization of active Pt^0^ sites. Similar morphology–driven interaction phenomena have been reported for other support materials, including CeO_2_, ZrO_2_, and Al_2_O_3_, where specific facets regulate electron density and catalytic efficiency [41, 48, 49, 50].

Consistent with these findings, the reactive {001} facets predominant on the proposed Pt/NS‐TiO_2_ catalyst facilitated stronger electronic interactions compared with the {101} facets of Pt/NR‐TiO_2_. This enhanced interaction was reflected in the more significant Ti 2p peak shift observed in XPS for Pt/NS‐TiO_2_, promoting electron donation from the support to the Pt nanoparticles. This resulted in a higher Pt^0^ fraction and improved dispersion, both of which were confirmed in our preceding analyses, compared with the Pt/NR‐TiO_2_. The Pt/NR‐TiO_2_, which was dominated by less reactive {101} facets, exhibited reduced electron transfer and consequently a lower Pt^0^ fraction and catalytic activity. Shao et al. further confirmed via DFT calculations that such facet‐dependent electronic interactions are crucial for stabilizing Pt^0^ active sites and optimizing catalytic performance [51, 52].

**FIGURE 8:**
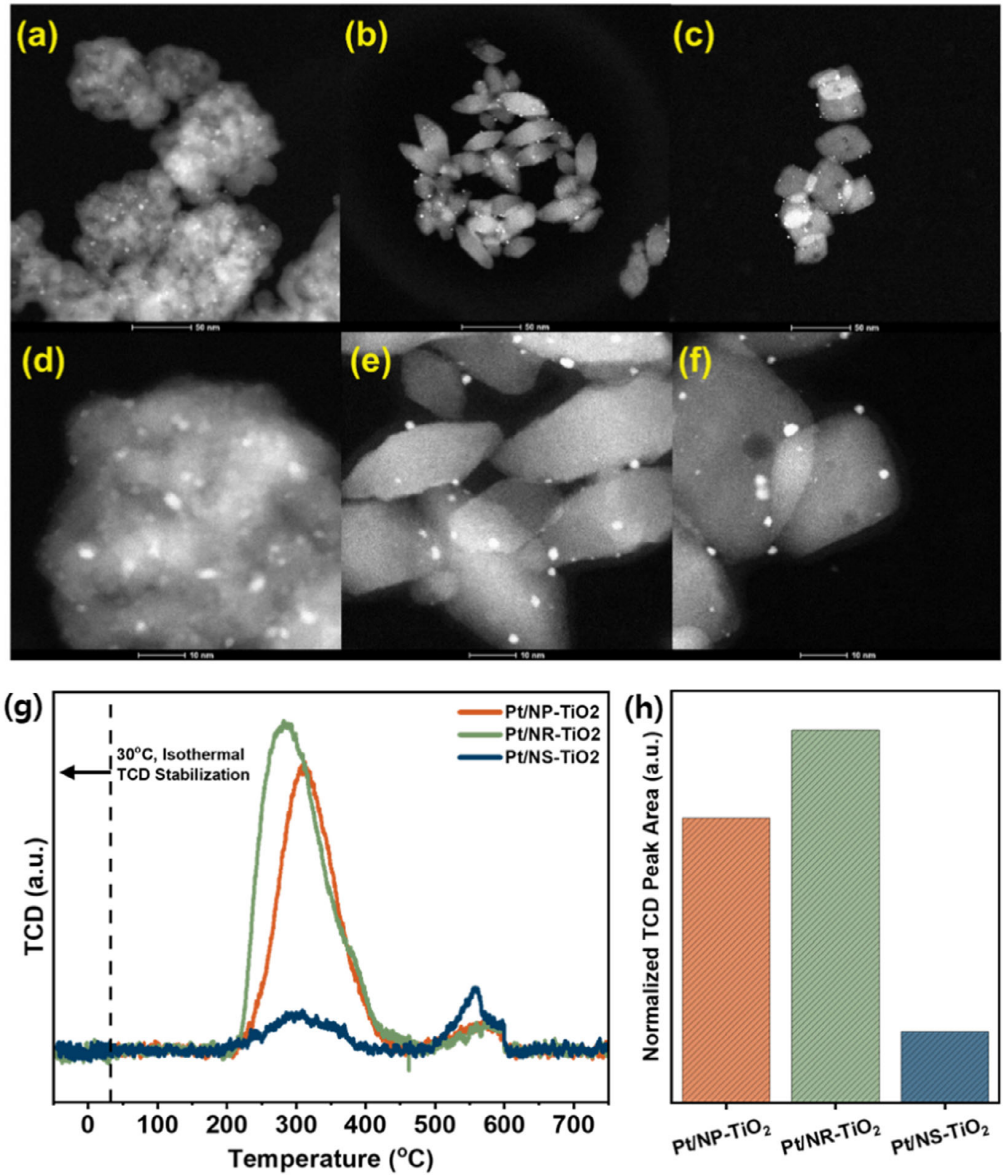
Post‐reaction characterization of the spent catalysts after 10 h of MCH dehydrogenation: TEM HAADF images of (a,d) Pt/NP‐TiO_2_ (b,e) Pt/NR‐TiO_2_, and (c,f) Pt/NS‐TiO_2_; (g) TPO profiles and (h) quantitative comparison of the total coke amount for each catalyst.

Figure 8 shows the HAADF–STEM images of the spent catalysts after 10 h on stream, providing insight into their structural features. All catalysts initially contained highly dispersed Pt clusters; however, some degree of Pt sintering was observed after reaction. The average Pt particle size increased in the order of Pt/NS‐TiO_2_ (d_TEM = 3.23 ± 0.04 nm) < Pt/NR‐TiO_2_ (3.51 ± 0.06 nm) < Pt/NP‐TiO_2_ (3.93 ± 0.07 nm). Among them, Pt/NS‐TiO_2_ maintained a more uniform particle size distribution after 10 h of reaction, indicating less pronounced particle growth over this period. This behavior is likely related to the predominantly exposed {001} facets of NS‐TiO_2_, which can induce stronger metal–support interactions than those in NR and NP morphologies [24]. Such enhanced SMSI is likely to contribute to improved Pt dispersion and may partially suppress sintering during the reaction [42].

Despite Pt growth, both the synthesized NR and NS supports retained their well‐defined morphologies. This initial stability results from the facet‐dependent surface energies and atomic arrangements; the {101} facets on the NR which possess lower surface energy lead to thermodynamic stability that helps maintain their morphological shape [53]. Meanwhile, the {001} facets on NS are kinetically stabilized, which preserves their morphology [54]. These findings highlight the superior structural robustness of the synthesized TiO_2_ nanostructures under the reaction conditions.

In addition to sintering, carbon deposition is another primary cause of catalyst deactivation. To investigate carbon deposition, TPO analyses were conducted on the spent catalysts after 10 h of MCH dehydrogenation, as shown in the figure below. The results clearly indicate that Pt/NS‐TiO_2_ accumulated significantly less total coke compared to Pt/NP‐TiO_2_ and Pt/NR‐TiO_2_. The TPO profiles also provide insight into the nature of the deposited carbon. The low‐temperature peak (~300°C) can be attributed to amorphous or weakly bound carbon species located near the Pt active sites, which are readily oxidized [55]. In contrast, higher‐temperature peaks are generally associated with more ordered, graphitic carbon species that require higher temperatures for oxidation [56]. Interestingly, although Pt/NS‐TiO_2_ exhibits the lowest total coke accumulation, a relatively higher fraction of high‐temperature oxidation peaks is observed, indicating the presence of more ordered carbon species. This suggests that the formation of amorphous carbon is effectively suppressed on the NS support, while the remaining carbon species tend to be more stable. This reduced formation of amorphous carbon is likely to alleviate active site blocking, thereby maintaining a higher number of accessible Pt active sites during the reaction. As a result, Pt/NS‐TiO_2_ exhibits enhanced catalytic performance due to improved active site availability.

**FIGURE 9:**
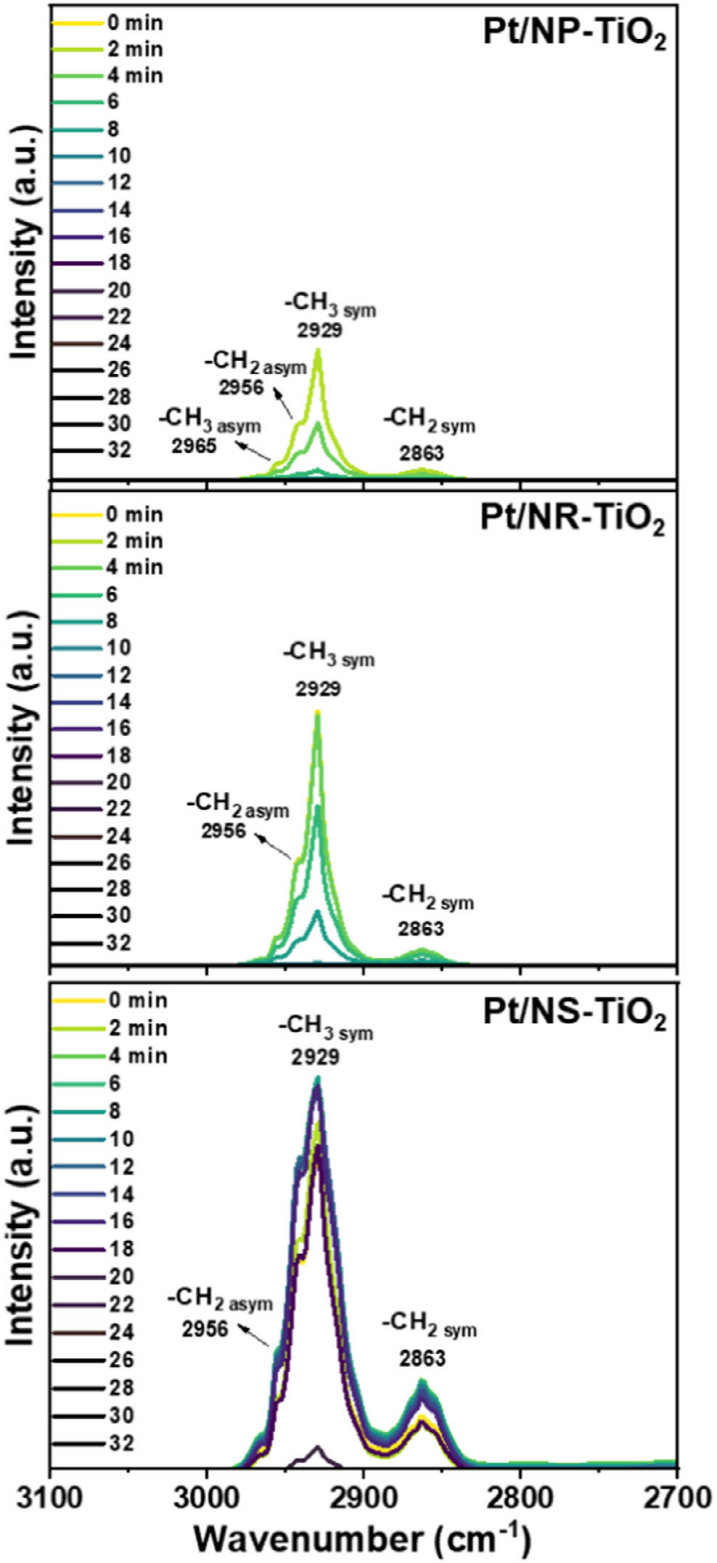
In situ DRIFTS results of MCH adsorption on Pt/TiO_2_.

As presented in Figure 9, in situ DRIFTS analysis of MCH adsorption was performed to establish the direct link between the measured surface acidity and the intrinsic affinity of the reactant for the active sites. The intensity of the sp^3^ C—H stretching peak (approximately 2950 cm^−1^), which served as a quantitative proxy for MCH adsorption capacity, followed the order NS >> NR > NP in its initial state. This result confirms the significantly higher MCH adsorption capacity of the Pt/NS‐TiO_2_ catalyst. The strong positive correlation between MCH adsorption capacity and DoDH catalytic activity is the central finding of this analysis, which confirms that the superior performance of Pt/NS‐TiO_2_ is quantitatively governed by its maximized ability to stabilize and activate MCH. Crucially, the quantitative MCH adsorption results derived from DRIFTS are in excellent agreement with the acid site density trend identified by NH_3_‐TPD. This combined evidence supports the conclusion that the {001} facet enhances surface acidity while simultaneously maximizing the affinity for key reactant molecules, which comprises superior activity by definition.

## 4 Conclusion

The structure–performance relationship of Pt/TiO_2_ catalysts was systematically elucidated via rational control of the morphology of the TiO_2_ support. Comprehensive characterization revealed that the exposed crystal facets of TiO_2_ play a crucial role in governing the physicochemical properties of the catalysts. In particular, the nanosheet‐supported catalyst (Pt/NS‐TiO_2_), predominantly exposing {001} facets, exhibits strong interfacial electronic interactions between Pt and the TiO_2_ support. These interactions stabilize a high fraction of catalytically active metallic Pt^0^ species and generate abundant weak‐to‐medium strength acid sites. In situ DRIFTS analysis further demonstrated that these physicochemical characteristics directly enhance the adsorption capacity for MCH, which underpins the superior dehydrogenation activity. Consequently, Pt/NS‐TiO_2_ delivers significantly higher intrinsic activity than catalysts supported on {101}‐faceted rhombic TiO_2_ (Pt/NR‐TiO_2_) and irregular nanoparticles (Pt/NP‐TiO_2_) while maintaining excellent morphological stability under reaction conditions. Overall, our findings establish a clear correlation between TiO_2_ facet exposure, interfacial electronic modulation of Pt, and reactant adsorption behavior. Therefore, this study provides a rational design strategy for efficient LOHC dehydrogenation catalysts.

## Author Contributions

**Yujung Jung**: formal analysis (lead), methodology (lead), validation (lead), visualization (lead), writing – original draft (lead). **Najwa Fatimah Somanjaya**: formal analysis (lead), investigation (lead), validation (lead), visualization (lead), writing – original draft (lead). **Hyunsik Hwang**: formal analysis (supporting), methodology (equal). **Yoondo Kim**: data curation (equal), formal analysis (supporting), investigation (supporting). **Hyangsoo Jeong**: formal analysis (supporting), investigation (supporting), writing review and editing (supporting). **Yongmin Kim**: conceptualization (supporting), formal analysis (supporting), writing review and editing (equal). **Keunsoo Kim**: formal analysis (equal), investigation (equal). **Minseok Bae**: conceptualization (supporting). investigation (equal), writing – original draft (supporting). **Suk Woo Nam**: conceptualization (supporting), methodology (supporting), writing original draft (supporting), writing – review and editing (supporting). **Muhammad Ridwan**: conceptualization (lead), supervision (lead), writing – original draft (supporting), writing – review and editing (lead). **Wangyun Won**: project administration (lead), supervision (lead), writing original draft (supporting), writing – review and editing (lead). **Hyuntae Sohn**: conceptualization (lead), funding acquisition (lead), project administration (lead), supervision (lead), writing – review and editing (lead).

## Funding

This work was supported by the Ministry of Trade, Industry and Energy (RS‐2024‐00435426 and RS‐2024‐00454737).

## Conflicts of Interest

The authors declare no conflicts of interest.

## Data Availability

The data that support the findings of this study are available from the corresponding author upon reasonable request.
